# Synthesis and Applications of Biopolymer Composites

**DOI:** 10.3390/ijms20092321

**Published:** 2019-05-10

**Authors:** Ana María Díez-Pascual

**Affiliations:** Department of Analytical Chemistry, Physical Chemistry and Chemical Engineering, Faculty of Sciences, Institute of Chemistry Research “Andrés M. del Río” (IQAR), University of Alcalá, Ctra. Madrid-Barcelona, Km. 33.6, 28805 Alcalá de Henares, Madrid, Spain; am.diez@uah.es; Tel.: +34-918-856-430

In recent years, there has been a growing demand for a clean and pollution-free environment and an evident target to minimizing fossil fuel. Therefore, a lot of attention has been focused on research to replace petroleum-based commodity plastics by biodegradable materials arising from biological and renewable resources. Different biopolymers, polymers produced from natural sources either chemically from a biological material or biosynthesized by living organisms, are also suitable alternatives to address these issues due to their outstanding properties including good barrier performance, biodegradation ability, and low weight. However, they generally present poor mechanical properties, a short fatigue life, low chemical resistance, poor long-term durability, and limited processing capability. In order to overcome these deficiencies and develop advanced materials for a wide range of applications, biopolymers can be reinforced with fillers or nanofillers (with at least one of its dimensions in the nanometer range) to form biocomposites or bionanocomposites. In particular, nanostructures can exhibit higher specific surface areas, surface energy, and density, compared to conventional microfillers, and can lead to materials with new and improved properties due to synergistic effects that are better than those arising from the simple rule of mixtures. Therefore, bionanocomposites are advantageous for a wide range of applications, such as medicine, pharmaceutics, cosmetics, food packaging, agriculture, forestry, electronics, transport, construction, and so forth. This Special Issue, with a collection of 17 research articles, provides selected examples of the most recent advances in the synthesis, characterization, and applications of environment friendly and biodegradable biopolymer composites and nanocomposites.

The most widely used biopolymers for the current development of biocomposites are poly(lactic acid) (PLA), cellulose esters, polyhydroxyalkanoates (PHAs), and starch-based plastics [1,2]. PLA is a fully renewable polymer that is both resorbable in the human body and biodegradable in composting plants. It presents biocidal activity because of its tendency to hydrolyze on the surface, producing lactic acid, and is one of the best alternatives to petroleum-based polymers in the packaging, agricultural, personal care, cosmetic, biomedical, and tissue engineering sectors [1,3,4]. A large number of studies have been devoted to extend its processability and the range of applications by reinforcing it with different nanofillers, including cellulose nanocrystals, chitin nanofibers, metal oxide nanoparticles, or clays [5,6]. In particular, the combination of PLA and chitin nanofibers represents a good opportunity for the preparation of bioplastic materials with improved structural and functional properties due to synergistic effects. However, it is difficult to attain a uniform dispersion of these nanofibers within the PLA matrix at the nanoscale level. In this regard, Coltelli et al. [7] used poly(ethylene glycol) (PEG), a biocompatible polymer, to prepare pre-composites that were subsequently added to PLA in the extruder to obtain transparent nanocomposites. The tensile properties did not show a reinforcing effect of up to 12 wt% chitin loading, albeit the nanocomposites maintained high values of elongation at break (>150%). This methodology is advantageous since it can be applied at an industrial level and does not modify the thermo-mechanical properties of plasticized PLA. This is in contrast to the results found upon the addition of diverse types of cellulose microfibers with different aspect ratios, where the stiffness increased with increased filler loading [8]. Some cellulose microfibers can be used without any compatibilization in order to reduce the final composite cost, increase the stiffness, and simultaneously promote the biodegradability of the materials.

An interesting nanofiller for PLA is potato pulp powder, utilized as a residue of the processing for the production and extraction of starch. It consists mainly of lignocellulosic fibers, starch, and proteins, and the cost of the raw material is low, which makes it very appealing for industrial application. In this regard, Righetti et al. [9] developed PLA/potato pulp biocomposites by extrusion followed by injection molding and characterized them in terms of processability and thermal, mechanical, and rheological properties. To make the processing easier, acetyl tributyl citrate (ATBC), derived from naturally occurring citric acid, was used as plasticizer and calcium carbonate was added in low percentages as an inert filler to facilitate the detachment of the injection-molded specimens. A slight drop in stiffness was found, compared to the neat matrix, together with a small reduction in ductility, since the potato pulp particles act as stress concentration sites and promote crack nucleation. Nonetheless, the lower viscosity of the biocomposites is an advantage for the material processing, which meets the requirements for rigid food packaging applications. The biomedical uses of PLA composites are also of great interest. Continuing the progress in this topic, Zhao et al. [10] developed PLA based-composite films, reinforced with stearic acid-modified MgO whiskers via a solution casting method, and studied their in vitro degradation properties and cytocompatibility. The degradation behaviour of the composites was found to increase with increasing MgO content and was pH-dependent. Furthermore, the cytocompatibility of the composites also increased considerably, which is beneficial for promoting cell proliferation and improving the matrix bioactivity.

PHAs are very interesting biopolymers. They are a family of polyesters of hydroxyalkanoic acids, synthetized by microorganisms in the presence of excess carbon and lack of essential nutrients. PHAs have thermoplastic properties similar to those of polypropylene, good mechanical properties, and excellent biodegradability in various ecosystems [11]. The most common PHAsare the homopolymer poly(3-hydroxybutyrate) (PHB) and its copolyester with hydroxyvaleric acid, poly(3-hydroxybutyrate-co-3-hydroxyvalerate) (PHBV), which are well suited for food packaging [12]. Despite their good properties and excellent biodegradability, their costs are relatively high (€7–12/kg) compared to other biopolymers, such as PLA (€2.5–3/kg), and has limited their use in the medical and pharmaceutical sectors. A lot of effort has been devoted to incorporate low-value nanomaterials into PHAs in order to reduce the cost of the final products. Thus, Cinelli et al. [13] incorporated waste wood sawdust fibers, a byproduct of the wood industry, into PHB via melt extrusion, using ATBC as plasticizer and CaCO_3_ as inert filler. The impact resistance of the composites increased notably with increased fiber loading. More importantly, the fibers accelerated the degradation of the polymeric matrix in soil. Hence, these composites are interesting in agriculture or plant nursery. With the aim ofimproving the performance of PHAs in terms of heat resistance, stiffness, and toughness, cellulose fibers and a thermoplastic polyurethane (TPU) have been added to PHBV [14]. To improve PHBV-cellulose interfacial adhesion, different compatibilizing agents were tested, including hexametylene diisocianate, an epoxy-functionalized styrene-acrylic oligomer, and triglycidyl isocyanurate. The diisocianate displayed the best compatibilization ability, with the uppermost values of elongation at break and toughness. This strategy can aid to solve some of the issues that these materials encounter in common applications.

On the other hand, cellulose is environmentally conscious, low-cost, strong, dimension-stable, non-melting, non-toxic, and can be derivatized to covalently append a wide range of biologically active molecules. In particular, Edwards et al. [15] compared the performance of a sensor designed with a nanocellulose aerogel transducer surface, derived from cotton, with cotton filter paper and nanocrystalline cellulose. X-ray crystallography, Michaelis–Menten enzyme kinetics, and circular dichroism were used to assess the structure/function relations of the peptide-cellulose conjugate conformation to enzyme/substrate binding and turnover rates. The aerogel-based sensor yielded the highest enzyme efficiency, ascribed to the binding of the serine protease to the negatively charged cellulose surface.

The interest towards nanoscale cellulose has increased extraordinarily over the last years, owed to its inherent mechanical properties, which are better than those of the source biomass material [16]. The combination of carbon nanotubes (CNTs) and cellulose results in a conductive nanocomposite network that can be used in a wide range of applications, including supercapacitor electrodes, electromagnetic interference shielding devices, and water and pressure sensors [17]. The properties of nanocellulose-CNT composites are affected by the quality of the CNT dispersion, the amount of defects, and the aspect ratio of the CNTs, as well as the strength of the CNT-cellulose interactions. The key challenge is to achieve a uniform and stable CNT dispersion. To attain such goal, Siljander et al. combined ultrasonication with the addition of surfactants [18] and found that there are a number of parameters that strongly affect the nanocomposite conductivity, such as surfactant type and concentration, sonication energy, and the film processing technique and the best performance was attained with the non-ionic surfactant Triton.

Natural rubber is another interesting non-toxic material derived from a renewable stock that has excellent physical properties and, due to its low price, is the elastomer most used in industry worldwide. Continuing the progress in this topic, Manaila et al. [19] developed environmental-friendly natural rubber/plasticized potato starch composites via peroxide cross-linking in the presence of trimethylolpropane trimethacrylate as a cross-linking co-agent. The influence of starch concentration on the mechanical properties, gel fraction and cross-link density, water uptake, structure, and morphology, before and after thermal degradation and natural ageing of the composites, were investigated. Plasticized starch loading up to 20 wt% was found to have a reinforcing effect on the matrix, and also favored its natural degradation; hence, starch can be considered as an interesting alternative to conventional fillers such as silica and carbon black.

Potato starch is a protein-rich polymeric by-product currently used in animal feed industries. Its combination with wheat gluten, also a protein-rich material, is interesting for the development of bio-based plastics [20]. However, these raw materials are difficult to process since their glass transition temperature (T_g_) is close to their thermal degradation temperature. Hence, chemical agents that reduce the T_g_ and broaden the processing window are required. Chemical additives such as NaOH create basic conditions for the proteins, resulting in changes of their secondary and supramolecular structures that lead to improved functional properties of the processed materials [21]. In particular, increasing the wheat gluten content in the composites was found to decrease the protein solubility and the Young’s modulus, albeit enabling the manufacture of films with good properties at a low pressing temperature (i.e., 130 °C), thereby contributing to a lower environmental foot-print due to a reduction of energy use [22]. Silk fibroin is another polymeric protein that has outstanding mechanical properties and a tunable biodegradation rate, due to its variable structures. Different fabrication methods can affect the structural transitions and physical properties of silk fibroin materials. In this sense, Liu et al. [23] investigated the variability of structural, thermal, and mechanical properties of two silk films (Chinese and Thailand B. Mori) regenerated from a formic acid solution, as well as their original fibers, using dynamic mechanical analysis (DMA) and Fourier transform infrared spectrometry (FTIR). Chinese silks were found to display a lower T_g_, a higher disorder degree, and better elasticity and mechanical strength. Further, as the calcium chloride content in the initial processing solvent increased, the T_g_ of the samples decreased while their disorder degree raised. These findings provide useful insight into the development of advanced protein biomaterials with different secondary structures.

Renewable polymeric materials, from vegetable or plant oils, can also be used as reliable starting material to access new products with a wide array of structural and functional variations [24]. Their abundant availability and relatively low cost make them industrially attractive for the plastics industry. Vegetable oils (soybean oil, castor oil, linseed oil, etc.) have been polymerized in the presence of various fillers and fibers, such as clays, inorganic nanoparticles, hemp, flax, jute or kenaf fibers, and so forth, leading to biocomposites that show significant improvements in their mechanical properties and thermal stabilities [25,26,27]. Cinnamon oil is also a highly interesting additive and it can be extracted from various parts of the cinnamon plant, such as leaf, bark, flower, and root. The major compounds in leaf and bark cinnamon oil are eugenol and trans-cinnamaldehyde, respectively, which present antioxidant and antimicrobial activities and can be added to alginate-based films to fabricate active packaging materials. In this regard, Baek et al. [28] added low amounts (up to 1 wt%) of cinnamon leaf and bark oils to Ecklonia cava alginate, in the presence of CaCl_2_, as a cross-linking agent. As the content of the oils increased, the tensile strength decreased, while the elongation at break increased. The antioxidant activities of the films with bark cinnamon were higher than those of films with leaf cinnamon. In contrast, the antimicrobial activities against Escherichia coli, Salmonella typhimurium, Staphylococcus aureus, and Listeria monocytogenes were better in the films with leaf cinnamon, corroborating that both types of films can be applied as new active packaging materials.

Another interesting biopolymer is chitosan, often obtained from the exoskeleton of crustaceans. It has very valuable properties, including biocompatibility, biodegradability, and antimicrobial activity. The production of chitosan products is difficult due to its insolubility in organic solvents, its ionic character in solution, and the formation of three-dimensional networks by strong hydrogen bonds [29]. However, a great breakthrough has been done with producing chitosan fibers. To improve fiber formation, Sanchez-Alvarado et al. [30] combined an anionic biodegradable poly(vinyl alcohol) (PVA) using the electrospinning technique. Different chitosan concentrations (0.5, 1, 2, and 3 wt%) were tested and the electrospinning parameters (syringe/collector distance, solution flow, and voltage) were optimized. Furthermore, the fibers were treated with ethanolic NaOH solution to make them chemically stable. On the other hand, the grafting of α-tocopherol succinate to the skeleton of glycol chitosan leads to an amphiphilic polymer that can form micelles suitable for the delivery of paclitaxel [31], a powerful anti-tumor drug extensively used in the clinical treatment of tumors. Micelles loaded with this anticancer agent showed good antitumor activities, in vitro and in vivo, and had advantages over commercially available formulations in terms of lower toxicity levels and a higher tolerated dose. Analogously, hydrophobic deoxycholic acid and folic acid (FA) have been used to modify chitosan, leading to another amphiphilic polymer that was a safe and effective carrier for the intravenous delivery of paclitaxel [32].

An alternative approach to produce biodegradable polymeric materials is the use of CO_2_. In this sense, Chen et al. [33] copolymerized CO_2_ with other monomers, propylene oxide (PO), and cyclohexene oxide (CHO) to synthesize random copolymers, di-block, and tri-block copolymers. Pyrolysis-gas chromatography/mass spectrometry and thermogravimetric analysis/infrared spectrometry techniques were applied to examine the thermal degradation behaviour of the polymers. The results showed that, in all cases, unzipping was the main degradation mechanism. The random copolymer showed a one-step decomposition with very high degradation temperatures. Hence, random copolymerization of CHO, PO, and CO_2_ seems to be a better way to improve the thermal stability of poly(propylene carbonate (PPC)–cyclohexyl carbonate than block copolymerization. Blends of PPC and polyester-based TPU have also been developed via melt compounding [34] and the compatibility, thermal, and mechanical properties, as well as the toughening mechanism of the blends, have been investigated via FTIR, differential scanning calorimetry (DSC), DMA, and tensile and impact tests. For these materials, FTIR revealed strong interfacial adhesion between the polymers, which resulted in more enhanced thermal stability and mechanical properties than the individual polymers. Moreover, the blends with 20 wt% polyurethane exhibited a brittle-ductile transition.

It is also interesting to examine the potential of these biopolymer composites from an application viewpoint, considering their properties and costs, as summarized in Table 1. For instance, the incorporation of about 20 wt% of potato pulp powder to PLA offers the possibility to markedly reduce the cost of PLA-based composites for common applications, like food packaging [9]. However, those based on PHB [13], PHBV [14] or comprising vegetable oils [28] are not cost effective in such applications and would only be used in active packaging, or for biomedical purposes like tissue engineering. On the other hand, rubber [19] and potato starch-based [22] composites are an interesting and relatively cheap alternative to petroleum-based plastics. It is important to note that the international market for biopolymers/bioplastics is still in its infancy. Nevertheless, owing to increasing prices of petrochemical feed stocks for plastics, along with growing environmental considerations, would pave the way for a bright future for these materials, including their biodegradable composites. These materials would be essential to realize and maintain a sustainable productive society that produces waste materials at a rate at which they can be reabsorbed by the environment.

What should we expect for the next years? It is clear that the field of biopolymer composites will continue growing with the incorporation of new nanofillers and the development of complex hybrid materials to be applied in a wider range of fields. For instance, a market study by Helmut Kaiser Consultancy has reported that the availability of bioplastics during the last decade has the potential to reduce the petroleum consumption for plastic by 15%–20% by 2025 [35]. The global bioplastics production capacity is set to increase from around 2.1 million tonnes in 2019 to 2.6 million tonnes in 2023. PLA and PHAs are driving this growth. The market is growing rapidly, since a large number of companies are entering it with newer innovations and applications in packaging, food services, agriculture, automotive, electronics, household appliances, and consumer goods. Europe is the largest bioplastic market, owed to limited crude oil reserves. The applications responsible for higher market growth are food and beverage packing, catering products, and bags. The research on biopolymer composites, although still in its initial stage, has shown their great potential to replace conventional composites based on petroleum derived plastics. However, some challenges should be tackled in the future, including appropriate fatigue life, improved capability forfiber forming and drawing, strict control of degradation time, longer operation life time, and comparable strength values to those of advanced composites (i.e., carbon fiber reinforced polymers, CFRPs) for high performance applications, to mention a few. In addition, to fully attain their potential at an industrial level, increased investment capital and well-developed government incentives are desired.

## Figures and Tables

**Table 1:** Summary of biopolymer composites properties, applications, and costs.

Matrix/Filler	Production Method	Properties	Applications	Cost/Kg(€)	Ref.
PLA/PEG/Chit	Extrusion	Low stiffness/High flexibility	Bone & dental implants food packaging	3.0–4.2	[7]
PLA/Cellulose	Extrusion/injection	Improved rigidity & biodegradability	Packaging, automotive industry, building	2.7–3.1	[8]
PLA/Potato pulp	Extrusion/injection	Low stiffness & ductility, good processability	Food packaging	2.4–2.7	[9]
PLA/MgO	Solution casting	Improved stability and bioactivity	Medical implants, tissue engineering, orthopedic devices	2.8–3.3	[10]
PHB/wood sawdust fibers	Extrusion	Improved degradation in soil	Agriculture or plant nursery	5.6–7.0	[13]
PHBV/TPU/cellulose	Extrusion/injection	Balanced heat resistance, stiffness, and toughness.	Food packaging tissue engineering	8.2–9.8	[14]
Nanocellulose/CNT	Cast molding	Good electrical conductivity	Supercapacitor, sensors	2.4–11.5	[18]
Rubber/potato starch	Roller mixing	Accelerated thermal ageing	Vibration isolators, shock mounts, electrical components	1.7–1.9	[19]
Potato starch/wheat gluten	Compression molding	Improved maximum stress & extensibility	Development of bio-based plastics	0.8–1.2	[22]
Alginate/cinnamon oil	Solution casting	Good antibacterial activity	Active packaging materials	7.3–8.2	[28]
PVA/Chitosan	Electrospinning	Good chemical stability	Drug delivery food packaging	1.5–1.8	[30]
PPC/TPU	Melt compounding	Good thermal stability & stiffness	Electronicpackaging applications	4.1–5.0	[34]
